# A 50-Year-Old Patient with Guillain–Barré Syndrome after COVID-19: A Case Report

**DOI:** 10.3390/medicina57080775

**Published:** 2021-07-29

**Authors:** Tomasz Chmiela, Michalina Rzepka, Ewa Krzystanek, Agnieszka Gorzkowska

**Affiliations:** 1 Department of Neurology, Faculty of Medical Sciences in Katowice, Medical University of Silesia, 40-752 Katowice, Poland; michalina.rzepka93@gmail.com (M.R.); ekrzystanek@sum.edu.pl (E.K.); 2 Department of Neurorehabilitation, Faculty of Medical Sciences in Katowice, Medical University of Silesia, 40-752 Katowice, Poland; agorzkowska@sum.edu.pl

**Keywords:** SARS-CoV-2, coronavirus, COVID-19, Guillain–Barré syndrome (GBS), acute inflammatory demyelinating polyneuropathy (AIDP), neuropathy

## Abstract

Severe acute respiratory syndrome coronavirus 2, or SARS-CoV-2, causes acute respiratory disease (coronavirus disease 2019; COVID-19). However, the involvement of other mechanisms is also possible, and neurological complications are being diagnosed more frequently. Here, we would like to present a case of a Polish patient with Guillain–Barré syndrome (GBS), after a documented history of COVID-19: A 50-year-old man, 18 days after the onset of COVID-19 symptoms, had progressive quadriparesis preceded by 1-day sensory disturbances. Based on the clinical picture, the results of diagnostic work-up including a nerve conduction study (ENG) that revealed a demyelinating and axonal sensorimotor polyneuropathy, and cerebrospinal fluid (CSF) analysis that showed albumin–cytological dissociation, an acute inflammatory demyelinating polyneuropathy was confirmed, consistent with GBS. Upon a therapeutic plasma exchange (TPE), the patient’s condition improved. The presented case of GBS in a patient after mild COVID-19 is the first case in Poland that has supplemented those already described in the global literature. Attention should be drawn to the possibility of GBS occurring after SARS-CoV-2 infection, even when it has a mild course.

## 1. Introduction

Severe acute respiratory syndrome coronavirus 2, or SARS-CoV-2, causes acute respiratory disease. By May 2021, COVID-19 had affected over 150 million people worldwide and accounted for over 3 million deaths [1]. The main manifestation of COVID-19 includes involvement of the respiratory system; however, the disease may also affect some other systems, including pathological findings in the nervous system [1,2]. The most common neurological disorders are olfactory and/or taste disorders, headache, or myalgia [2]. However, COVID-19 can also be manifested as an ischemic stroke, intracerebral hemorrhage, status epilepticus, acute disseminated encephalopathy, and as various symptoms related to a peripheral nerve injury [1,2,3].

Guillain–Barré syndrome (GBS) is a rare, potentially fatal, autoimmune peripheral nervous system disease, which is usually preceded by an upper respiratory or gastrointestinal infection [4]. GBS is a neuroinflammatory disease with a global incidence of 1–2 per 100,000 person-years.

The incidence increases with age, and unlike other autoimmune diseases, it is more prevalent in men [5]. GBS represents a heterogeneous syndrome with several variants. The most frequent form is acute inflammatory demyelinating polyneuropathy (AIDP), whereas other variants are acute motor axonal neuropathy (AMAN), acute motor and sensory axonal neuropathy (AMSAN), Miller Fisher syndrome (MFS), acute sensory neuropathy, acute autonomic neuropathy, and pharyngeal–cervical–brachial paresis [4].

In the pathogenesis of GBS, the participation of different viral infections, such as influenza virus, cytomegalovirus (CMV), Epstein–Barr virus (EBV), Zika virus, and MERS virus (Middle East respiratory syndrome), has been considered [4,6,7]. The incidence of GBS may increase during outbreaks/pandemics of infectious diseases, as was previously observed during the Zika virus epidemic, in French Polynesia, in 2013 [8]. At the time of writing, there have been approximately 100 reports of GBS associated with COVID-19, but so far, none have originated from Poland. Based on the analysis of 94 known cases of GBS, related to COVID-19, it was found that the course of disease does not differ significantly from the classic form of GBS [9]. However, the relationship between SARS-CoV-2 and GBS still remains under investigation. Most of the cases described in the medical literature do not exclude the possibility of causes of GBS other than the SARS-CoV-2 infection, whereas a recent epidemiological study by Keddie et al. even suggests a complete lack of association between COVID-19 and GBS [10].

This article discusses a case of a 50-year-old male with GBS post-COVID-19. To the best of our knowledge, this is the first documented case of post-COVID-19 GBS in Poland. The presented case aims to complement the current picture of COVID-19 neurological complications.

## 2. Case Presentation

A 50-year-old patient was admitted to the Department of Neurology due to progressive muscle weakness in all four extremities and progressive sensory disturbances. The onset of symptoms in the form of paresthesia of the tongue, toes, and fingers occurred 3 days before his admission. The next day, the symptoms were accompanied by lower limb muscle weakness (which had progressed further in the following days). On the third day, troublesome paresthesia affected his entire limbs and the torso. In addition, on the second day, a peripheral seventh nerve palsy on the left side occurred, visual acuity deteriorated, and sphincter dysfunction (uncontrolled urination) occurred. The patient had undergone a SARS-CoV-2 infection (confirmed by an RT-PCR test) 17 days before the onset of GBS symptoms. In the course of SARS-CoV-2, the patient initially experienced musculoskeletal pain and low-grade fever (below 37.5 °C), severe headaches, and fever up to a maximum of 38 °C in the following days (mainly at night). Respiratory rate, heart rate, and blood pressure were normal. Therapy involved the use of paracetamol on an ad hoc basis. The symptoms subsided completely after two weeks. On the day before the onset of GBS symptoms (on the 16th day after the onset of the first COVID-19 symptoms), the patient had an RT-PCR test for SARS-CoV-2, which revealed a negative (non-reactive) result. In the past, the patient had not been treated for any chronic diseases.

**Figure 1:**
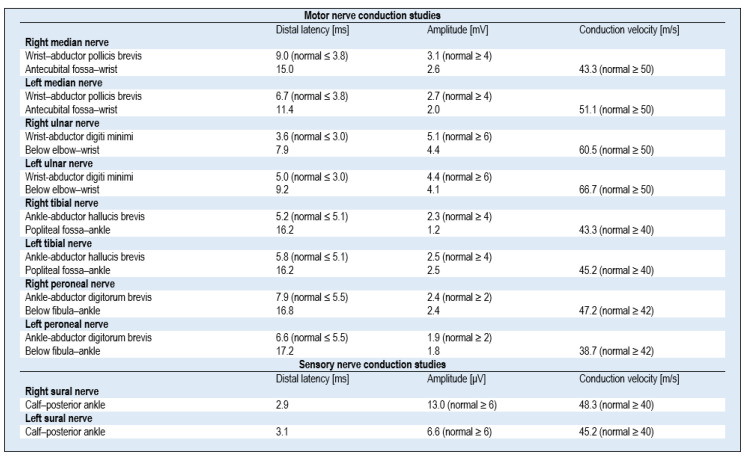
Nerve conduction study results for a 50-year-old man with post-COVID-19 Guillain–Barré syndrome performed 3 days after symptom onset.

Based on his neurological examination (upon his admission to the Department of Neurology), apart from confirming the symptoms as above, including four-limb paresis (on the Lovett scale—4), the patient was found to have decreased muscle tone in all four extremities, poorly expressed deep reflexes of the upper extremities, no reflexes evoked in the lower extremities, distal sensory impairment in all four extremities, and paraparetic gait. Walking on heels or toes was impossible. Computed tomography (CT) of the head excluded an acute ischemic or hemorrhagic stroke, and other focal cerebral lesions. A nerve conduction study (NCS) performed on the first day of hospitalization revealed mild features of sensorimotor radiculopolyneuropathy (Figure 1) The study of the cerebrospinal fluid (CSF) performed on the first day of hospitalization revealed albumin–cytological dissociation—cytosis 2/3, protein level 177.9 mg/dL (laboratory reference range: 15–45 mg/dL). On day 1 of hospitalization, the symptoms progressed further, including limb paresis that shifted to grade 4 on the Medical Research Council (MRC) scale, significantly impaired gait, increasing sensory disturbances involving the entire extremities and the trunk with a sensory level Th10, and gastrointestinal motility disorders.

**Figure 2:**
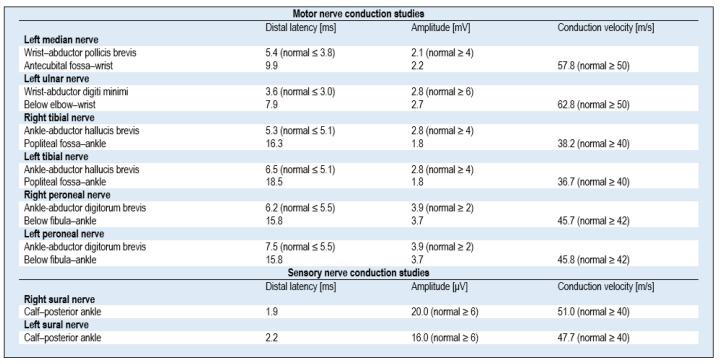
Follow-up nerve conduction study results for a 50-year-old man with post-COVID-19 Guillain–Barré syndrome after plasmapheresis (12 days after symptom onset).

The patient was initially diagnosed in the clinical setting, and his subsequent electrophysiological examination, and an analysis of the CSF, confirmed the clinical evaluation of the GBS (classic form) diagnosis, consistent with a post-infection manifestation of COVID-19. The patient met The National Institute of Neurological Disorders and Stroke (NINDS) Diagnostic Criteria for GBS; other causes of paresis were excluded. The patient qualified for plasmapheresis (PTE) therapy, and a series of five treatments (every other day) was performed in the intensive care unit. After the third PTE session, the patient reported improved gait and a partial resolution of sensory disturbances. On the following day, the patient was able to move without a walker. Immediately after the completion of PTE treatment, the patient had persisting discrete paresis of the seventh nerve on the left side, slight grade IV degree tetraparesis with no significant influence on gait efficiency, no sensory impairment, unstable unpleasant trunk paresthesia (especially at night, which awakened the patient), and no gastrointestinal motility disorders. Follow-up examinations of nerve conduction revealed features of demyelinating and axonal sensorimotor polyneuropathy (Figure 2). It should be noted that the first examination was performed very early, and at that point, typical changes, characteristic of GBS (detailed parameters are presented in Figure 1 and Figure 2), were not yet revealed.

**Figure 3:**
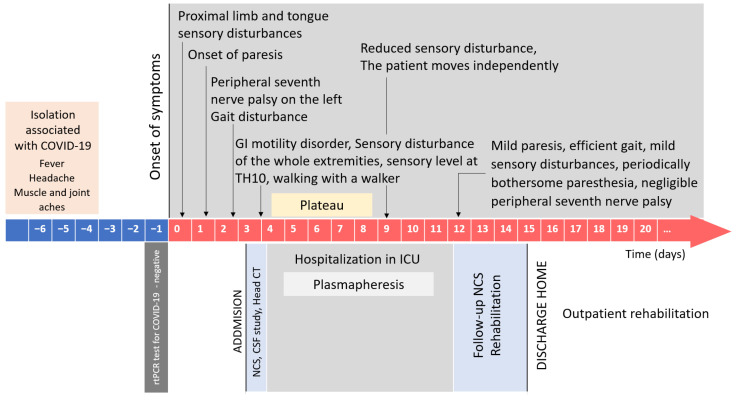
A course of the disease in a 50-year-old patient with Guillain–Barré syndrome after COVID-19.

On day 12 of hospitalization (15 days from the onset of GBS symptoms), the patient was discharged home in a good general condition, with a mild paresis of the lower extremities, not affecting his walking performance, and with dysesthesia, involving the fingers and toes; otherwise, there were no neurological deficits. Figure 3 shows the course of the disease. One month after discharge from the Neurology Department, the remaining symptom included only a non-troublesome finger paresthesia of the right limbs. Otherwise, there were no sensory disturbances or loss of muscle strength. Neurological examination revealed a weakening of deep knee and ankle reflexes (GBS disability score = 1).

## 3. Discussion

The literature suggests that the novel coronavirus may have neurotrophic and neuroinvasive features [9,11]. The mechanism of GBS development in COVID-19 is not fully known, although there are a number of theories as to how the infection affects the nervous system. The first potential variant is the immune mechanism. COVID-19 stimulates the cells of the immune system, inducing a humoral and cellular response; it causes the formation of antibodies and a considerable increase in the concentrations of pro-inflammatory cytokines, such as interleukin-6 (IL-6) and tumor necrosis factor-alpha (TNF-α), resulting in an intense immune response, the so-called cytokine storm [12,13,14]. Due to molecular mimicry, antibodies formed after SARS-CoV-2 infection cross-react with neuronal antigens, inducing an immune response directed at myelin and peripheral nervous system axons, and causing acute axonal and/or demyelinating neuropathy [4]. The post-infectious immune mechanism of GBS is highly probable, considering the observed—as in the presented case—improvement after TPE or intravenous immune globulin (IVIG) treatment and the difference in the time of COVID-19 infection and the development of GBS [15,16]. The second less likely mechanism is the rare phenomenon of parainfection [12]; however, the absence of SARS-CoV-2 RNA in the cerebrospinal fluid, noted by other researchers, may rule out both parainfectious inflammation and direct neuronal damage [16].

Herein, we described a case of progressive quadriparesis in a post-COVID-19 patient with a diagnosis of the classic form of GBS. Abdullahi et al. conducted a systematic review of 83 cases of GBS patients after SARS-CoV-2 infection, which revealed that GBS had preceded COVID-19 in only two cases. Therefore, attention should be drawn to the exclusion of COVID-19 infection in any patient with GBS during the current pandemic [17]. In the presented case, the incidence of COVID-19 does not raise any doubts; the disease was confirmed with the real time polymerase chain reaction test (RT-PCR), and before the onset of GBS, the symptoms of COVID-19 completely subsided, and the patient obtained a negative RT-PCR test result.

In the presented case, the ENG study revealed features of mixed demyelinating and axonal polyneuropathy. In the study by Abu-Rumeileh et al., which comprised an analysis of 73 cases, it was found that the classic form of GBS, with sensorimotor presentation and acute inflammatory demyelinating polyneuropathy, was the most frequently described variant (51/73, 70% of cases) [18]. GBS symptoms developed on average 14 days from the onset of symptoms of COVID-19 infection [16,18]; in our patient, this time was 18 days. Usually—as in the presented case—patients experienced fever, cough, and other symptoms of respiratory tract infections, such as dyspnea [3]. In previous publications, the results of the nerve conduction study were most often typical for the demyelinating form of GBS, and it was found in 77.4% (48/62) of patients, whereas 14.5% (9/62) of cases were diagnosed with the axonal subtype of GBS, and 8.1% (5/62) with the mixed subtype [18]. In the presented case, the ENG study revealed features of mixed demyelinating and axonal polyneuropathy.

Existing CSF case studies have displayed albumin–cytologic dissociation (ACD). In existing case reports, 71.2% (42/59) of the cases reported ACD with a mean protein concentration of 100 mg/dL (in our study, it was 177.9 mg/dL) and no SARS-CoV-2 RNA was detected in the CSF in any of the patients [18]. In our case, CSF was not examined for SARS-CoV-2 RNA due to low suitability, as confirmed by previous research. The most common findings in biochemical and serological tests were leukopenia and an increased level of C-reactive protein (CRP) [18]. In the presented case, CRP was slightly increased to 5.5 mg/dL (laboratory reference range: >5 mg/dL); no leukopenia was found.

From the cases described in the literature, the majority of patients (85.7%; 60/70) were treated with IVIG or TPE, and glucocorticosteroids (GCS)—it should be noted that GCS are not recommended as a therapeutic option for GBS—were administered in 10 patients (10/70; 14.3%) [18]. Mechanical ventilation or non-invasive respiratory support was required in 21.4% (15/70) of patients [18]. A total of 72.1% (49/68) of patients with post-COVID-19 GBS achieved a partial or complete remission, whereas 5% (4/68) died [18]. In the study by Aladawi et al., which reviewed 109 cases of post-COVID-19 GBS, 40/99 patients required intensive care treatment, 33.3% (33/99) required mechanical ventilation, and 6.1% (6/99) of patients died [19]. A worse course of GBS was observed in older patients who had a more severe course of COVID-19 infection. In the described case, the risk factor for GBS was only the patient’s sex (GBS is more prevalent in men) [4,19]. Moreover, the relatively young age of the patient, the mild course of COVID-19, and the prompt treatment could positively affect the course of GBS.

In the study by Aladawi et al., the dominant variant, as in our case, was the classic sensorimotor form (64/99; 64.5%) in NCS AIDP (59/77, 76.6%). In half of the cases (50.5%; 50/99), the concentration of antiganglioside antibodies (found in autoimmune neuropathies and often associated with GBS subtypes) was assessed, but merely seven cases were positive (7/50; 14%) [20]. In the presented case, no antibodies reacting with the recombinant antigen gangliosides GM1, GD1b, and GQ1b were found.

The presented case has many features in common with those described previously in the literature. These features include the sensorimotor AIDP GBS, which most frequently occurs simultaneously with COVID-19 infection [16]. Moreover, we did not detect antiganglioside antibodies, the presence of which more often correlates with the axonal type of GBS than with the demyelinating type, and their prevalence in patients with GBSassociated with COVID-19 is also low [16,21]. To date, most patients have been treated with IVIG, and in the presented case, we used TPE. Our patient showed a good response to treatment after TPE, almost complete relief of symptoms, and a good prognosis after being discharged from the hospital, which may be associated with a mild course of COVID-19 infection. A previous COVID-19 infection with severe respiratory symptoms favors a worse prognosis; however, GBS increases the risk of respiratory failure and the need for mechanical ventilation due to possible weakening of the respiratory muscles [22]. The available treatment options for GBS increase the chance of clinical improvement. In most of the cases described so far (approximately 2/3), post-COVID-19 GBS achieved a good result (GBS disability score of ≤2); a worse course was most often associated with old age [18,23]. However, long-term prognosis is still uncertain. In the presented case, only minimal sensory symptoms (non-troublesome finger paresthesia of the right limbs) were present one month after discharge from the neurology department.

Despite the increasing number of case reports, the prevalence of post-COVID-19 GBS is unknown. The most recent meta-analysis conducted in December 2020 found the prevalence of GBS to be 0.15% among the entire studied population of COVID-19 patients (15 cases of GBS per 100,000 SARS-CoV-2 infections) [16]. That said, among neurological patients hospitalized due to COVID-19, the prevalence of GBS varied from 0.42% (5/1200) to 10.3% (6/58) [24,25].

This case study aims to draw attention to the possibility of acute post-infectious SARS-CoV-2 polyneuropathy and to indicate that even a relatively mild course of COVID-19 may be associated with this severe neurological complication.

## Data Availability

The data presented in this study are available on request from the corresponding author.
